# Extremofiles 2.0

**DOI:** 10.3390/microorganisms9040784

**Published:** 2021-04-09

**Authors:** Ricardo Amils, Felipe Gómez

**Affiliations:** 1 Centro de Astrobiología (INTA-CSIC), Torrejón de Ardoz, 28850 Madrid, Spain; gomezgf@cab.inta-csic.es; 2 Centro de Biología Molecular Severo Ochoa (CSIC-UAM), Universidad Autónoma de Madrid, Cantoblanco, 28049 Madrid, Spain

The exploration of extreme environments has led to the discovery of numerous environments that were, until recently, considered uninhabitable [1]. This, for several reasons, some, fundamental and related with the search for the limits of life [2], and others, more pragmatic and focused on the biotechnological potential of extremophiles, has sparked a marked growth of interest in the ecology of extreme environments [3]. The 2019–2020 version of Extremofiles 2.0, a Special Issue of *Microorganisms* devoted to extremophiles, has gathered eleven papers dealing with different aspects of microorganisms that thrive in extreme environments: five on halophiles [4,5,6,7,8], three on acidophiles [9,10,11], one on thermophiles [12], one on psycrophiles [13] and one on metal resistant microorganisms [14].

An important issue in this area of research is the biodiversity identified in different extreme environments. Maltman et al. compiled the current research on bacterial tellurite resistance, focusing on bacteria with a high level of resistance to this metalloid inhabiting extreme environments [14].

Liu et al. analyzed the complex eukaryotic community in the world’s deepest marine blue hole in the South China Sea, where significant differences were observed at different depths, and the most abundant microalgae assemblages detected were Dinophyceae at 10–20 m water column [4].

Leoni et al. analyzed the microbial communities in nine ponds with increasing salt concentrations from Margherita di Savoia Saltern (Italy), the largest athalasohaline saltern in Europe. They observed *Salinibacter* as the most abundant genus, followed by the archaeal *Halocuadratum* and *Natronomonas* [7].

Gris et al. characterized the microbial community in the Euganean thermal muds (Italy), detecting a stable cyanobacterial population dominated by one species of *Phormidium*, for which the complete genome sequence is reported [12].

Ayala-Muñoz et al. analyzed the diversity of an acidic, meromictic pit lake in the Iberian Pyrite Belt, Cueva de la Mora, in which Eukaryotes, predominantly *Coccomyxa*, dominated the upper layer, while Archaea, predominantly Thermoplasmatales, dominated the deep layer, and a combination of bacteria and eukaryotes were abundant in the chemocline [11].

Finally, Plugge et al. characterized the efficiency of a gas-lift bioreactor in which H_2_/CO mixtures instead of pure H_2_ were used. The addition of CO marginally affected the microbial community; over time acetate production increased and acetogenesis became the dominant process [8].

More specific questions were addressed by the rest of the authors. Vega et al. reported on the halotolerant bacterium *Staphylococcus equorum* EN21’s promotion of plant growth by attenuating the virulence of phytopathogens through quorum quenching [5].

Sampedro et al. reported the role of chemotaxis in the colonization of the halophilic bacteria, *Halomonas anticariensis* FP35T, on *Salicornia hispanica* plants, the role of oleanolic acid as a chemoattractant and the enhanced positive effects of strain FP35T on the development of the plant [6].

Avila-Jimenez analyzed the structure of taxonomic and functional gene distribution across Artic and Antartic locations and observed that, although taxonomic diversity differed significantly between locations, functional genes were distributed evenly throughout bacterial networks as well as across different geographic locations, which could have implications for ecological resilience in the case of rapid or sudden environmental changes [13].

Sánchez-España et al. found a natural attenuation of acidity and toxic metal concentrations toward the bottom of two meromictic, oligotrophic acidic mine pit lakes, Filón Centro and La Zarza, both in the Iberian Pyrite Belt. Analysis of the correspondent microbial diversity showed this to be the consequence of the precipitation of metal sulfides due to the production of biogenic sulfide.
